# Long-term administration of *Tetragenococcus halophilus* No. 1 over generations affects the immune system of mice

**DOI:** 10.1371/journal.pone.0267473

**Published:** 2022-04-26

**Authors:** Kunihiko Kotake, Toshihiko Kumazawa, Takahiro Adachi

**Affiliations:** 1 Ichibiki Co., Ltd., Nagoya, Japan; 2 Department of Precision Health, Medical Research Institute, Tokyo Medical and Dental University, Tokyo, Japan; University of South Florida, UNITED STATES

## Abstract

Japanese people have been consuming miso soup over generations; it is beneficial for health and longevity. In this study, *Tetragenococcus halophilus* No. 1 in miso was found to possess salient immunomodulatory functions. Recently, we also demonstrated its effect on boosting immunological robustness. Although the consumption of miso is suggested to affect health over generations, such a long-term experiment has not been conducted until now. Thus, we evaluated the effects of miso-derived *T*. *halophilus* No. 1 over generations on the immune system of mice. As the generations increase, the proportion of germinal center B cells tends to increase. Furthermore, we found that CD4^+^ T cells expressing CD69, an activation marker, were increased in the third generation of mice. In addition, the proportion of follicular helper T cells and regulatory T cells tended to increase. Among the subsets of CD4^+^ T cells in the fourth generation, effector T cells and effector memory T cells tended to increase. In contrast, central memory T cells and naive T cells decreased. Moreover, autoimmunity was suppressed by long-term administration of *T*. *halophilus* No. 1. Based on these findings, we believe that the long-term administration of *T*. *halophilus* No. 1 over generations promotes immune activation and tolerance and enhances immunological robustness.

## Introduction

The Japanese have a high life expectancy, possibly owing to their diet. A study investigating Japanese dietary habits and survival reported that people who consumed Japanese food frequently had a lower risk of death and a longer survival time. Japanese foods include miso soup in addition to rice, fish, and pickles [1]. Miso is a traditional Japanese fermented food item made by fermenting microorganisms, such as *Aspergillus*, lactic acid bacteria, and yeast, with salt and soybean or other grains, such as rice and wheat, as raw materials. The Japanese have been consuming miso, mainly in the form of miso soup, for a long time.

Based on the findings of some cohort studies, miso soup was found to reduce the risk of death in patients with gastric cancer [2, 3] and the risk of breast cancer [4]. Furthermore, animal studies have shown that miso has protective effects against stroke [5], visceral fat accumulation when combined with exercise [6], and elevation in blood pressure [7, 8]. The consumption of miso has also been found to reduce the risk of early preterm birth [9]. Thus, miso has been proposed to have different health benefits. The consumption of miso soup could be linked to the overall health and life span of Japanese people. Furthermore, we showed that immunological robustness is enhanced by the consumption of miso [10]. We also identified *Tetragenococcus halophilus* No. 1 derived from miso as a potent immunomodulator [11]. Furthermore, in a study on the visualization of Ca^2+^ signaling in intestinal epithelial cells *in vivo*, the response of lactic acid bacteria was found to be similar to miso [10, 12]. Based on these findings, we believe that some components in miso affect its constitution over generations and that the main ingredient may be lactic acid bacteria. However, no studies have examined the effects of lactic acid bacteria intake over generations. Therefore, we examined whether long-term continuous intake of lactic acid bacteria derived from miso affects the immune system over generations.

## Materials and methods

### Ethics statement

All mice were maintained under specific pathogen-free conditions following the animal care guidelines of Tokyo Medical and Dental University. The Tokyo Medical and Dental University (approval number A2019-207C4) approved all animal experiments.

### Bacteria

*T*. *halophilus* No. 1 isolated from miso was prepared [11]. Because heat generally kills the lactic acid bacteria in miso soup, sterilized lactic acid bacteria were used for the evaluation. It has also been reported that heat-killed *T*. *halophilus* No. 1 used in the experiment affected the immunity of mice [11].

### Mice

C57BL/6 mice (8 weeks old) were fed either CE-2 (CLEA Japan) as a control diet or CE-2 containing 1% heat-killed *T*. *halophilus* No. 1 for 2 weeks for short-term administration testing.

For long-term administration over generation testing, male and female C57BL/6 mice of the exact parental origin were divided into two groups. One group was fed CE-2 (CLEA Japan) as a control, and the other group was fed CE-2 containing 1% heat-killed *T*. *halophilus* No. 1. The progeny produced was designated as the first (F1) generation in each group. After continued administration of the abovementioned diet, the second (F2), third (F3), and fourth (F4) generations of mice were obtained. At 16 weeks of age, all mice were analyzed.

### Flow cytometry

A MACSQuant Flow Cytometer (Miltenyi Biotec) was used to analyze whether the cells utilized the following specific antibodies: Violet Fluor^™^ 450-conjugated anti-B220 (Tonbo Biosciences); Alexa Fluor 647-conjugated anti-CD4 (BD Pharmingen); Brilliant Violet 510^™^-conjugated anti-CD4 (BioLegend); Brilliant Violet 510^™^-conjugated anti-CD8a (BioLegend); Alexa Fluor 647-conjugated anti-GL7 (BioLegend), phycoerythrin (PE)-conjugated anti-CD62L (BD Biosciences); allophycocyanin (APC)-conjugated anti-CD44 (eBioscience); APC-conjugated anti-CD86 (Tonbo Biosciences); PE-conjugated anti-CD69 (BioLegend); PE-conjugated anti-PD-1 (Tonbo Biosciences); Brilliant Violet 421^™^-conjugated anti-C-X-C chemokine receptor type 5 (BioLegend); Alexa Fluor 647-conjugated anti-inducible T cell co-stimulator (BioLegend); eFour450-conjugated anti-CD127 (eBioscience); PE-conjugated anti-CD25 (Tonbo Biosciences); and APC-conjugated anti-CTLA4 (Tonbo Biosciences). In addition, dead cells were excluded by propidium iodide staining. FlowJo (FlowJo, LLC) was used to conduct data analysis.

### Measurement of serum immunoglobulin (Ig) levels

As previously described, the serum immunoglobulin levels were measured [13] using an enzyme-linked immunosorbent assay (ELISA). Approximately 10 μg/ml of sonicated herring sperm DNA (Sigma-Aldrich) was coated on ELISA plates. The antibodies alkaline phosphatase-conjugated anti-IgM and anti-IgG (SouthernBiotech) were used.

### Statistical analyses

The experimental data were represented as the mean ± standard deviations (SD). Statistical significance was analyzed using a two-tailed Student’s *t*-test for unpaired data for two groups and the Tukey–Kramer test for three or more groups.

## Results

### Long-term administration of *T*. *halophilus* No. 1 over generations activates CD4^+^ T cells

*T*. *halophilus* No. 1, as previously reported, eased the upregulation of activation markers, such as CD86 and CD69, on B cells and T cells *in vitro* [11]. First, mice were fed diets with or without *T*. *halophilus* No. 1 for 2 weeks. To examine the changes caused by short-term administration, mice spleen cells were analyzed. No difference was observed in the ratio of activation markers on B cells and T cells (S1 Fig).

**Fig 1:**
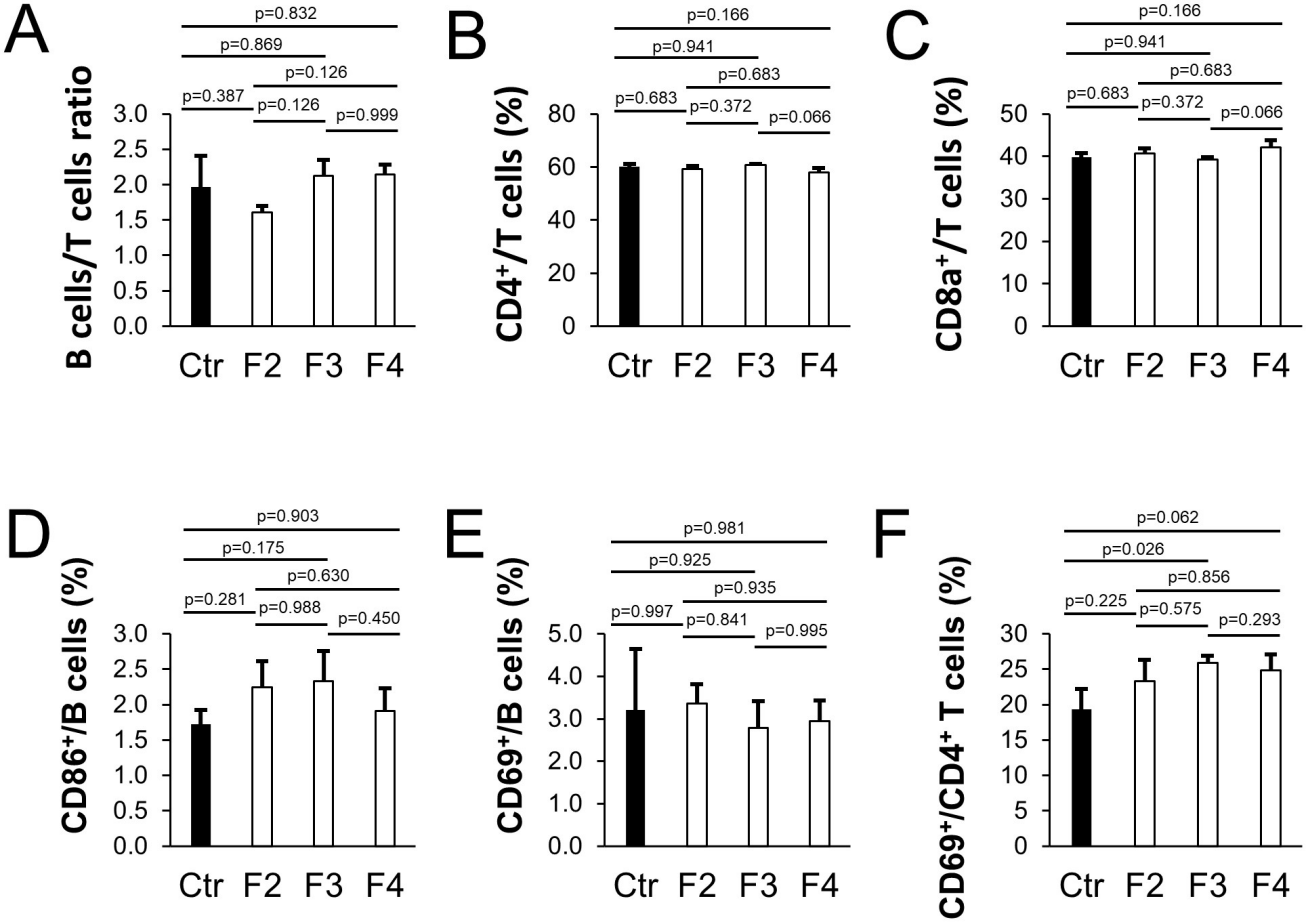
Intergenerational effects of *T*. *halophilus* No. 1 on lymphocytes in spleen. We examined the proportion of B cells and T cells in the spleen of mice. Mice (n = 4) were fed diets with or without 1% *T*. *halophilus* No. 1 until 16 weeks of age. Then, the spleen samples were obtained and analyzed using flow cytometry. Flow cytometry plots are shown in S2 Fig. For the control, the F2 mice fed control diet were used. (A) The ratio of B cells (B220^+^) and total T cells (CD4^+^ T cells and CD8a^+^ T cells in total). (B) The proportion of CD4^+^ among total T cells. (C) The proportion of CD8a^+^ among total T cells. (D) The proportion of CD86^+^ among B cells. (E) The proportion of CD69^+^ among B cells. (F) The proportion of CD69^+^ among CD4^+^ T cells. Bars indicate the mean ± SD. Based on the Tukey–Kramer test, P-value was found to be relative to the control.

Next, the effects of *T*. *halophilus* No. 1 on the spleen cells of mice that were continuously fed and passaged up to the F4 generation were investigated. The proportion of B and T cells and B and T cell activation markers in spleen cells from the F2, F3, and F4 generations was assessed. There was no change in the ratio of B and T cells in each generation (Fig 1A–1C). Furthermore, although no noticeable difference was observed in the proportion of B cell activation markers, CD86^+^ B cells tended to increase in the *T*. *halophilus* No. 1-fed group. In the F3 and F4 (Fig 1D–1F) generations, the proportion of activation markers in CD4^+^ T cells increased. These results suggest that long-term administration of *T*. *halophilus* No. 1 in mice over generations enhanced the activation of CD4^+^ T cells.

### Long-term administration of *T*. *halophilus* No. 1 over generations affects immune cells in terms of both activation and suppression

**Fig 2:**
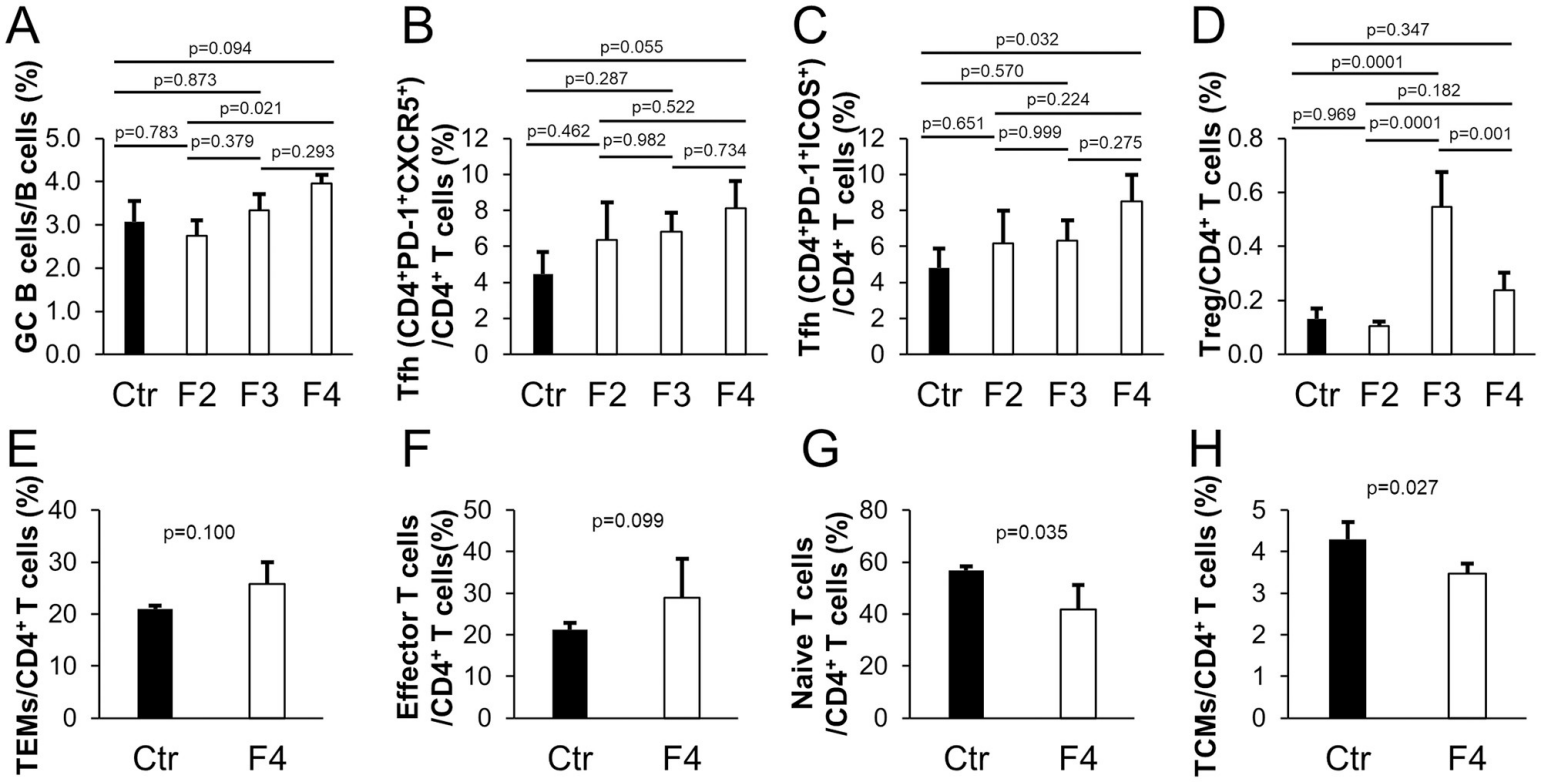
Intergenerational effects of *T*. *halophilus* No. 1 on B cells and T cells. We examined the proportion of B cell and T cell subsets in mice spleen. Flow cytometry plots are depicted in S3 Fig. (A) The proportion of GC B cells (B220^+^GL7^+^) among B cells. (B) The proportion of Tfh cells (CD4^+^PD-1^+^CXCR5^+^) among CD4^+^ T cells. (C) The proportion of Tfh cells (CD4^+^PD-1^+^ICOS^+^) among CD4^+^ T cells. (D) The proportion of Treg cells (CD4^+^CD127^−^CD25^+^CTLA4^+^) among CD4^+^ T cells. In (E)–(H), the proportion of subsets of CD4^+^ T cells in the F4 mice spleen was examined. The F3 generation data were used for the control. (E) The proportion of TEMs (CD44^high^CD62L^−^) among CD4^+^ T cells. (F) The proportion of effector T cells (CD44^low^CD62L^−^) among CD4^+^ T cells. (G) The proportion of naive T cells (CD44^low^CD62L^+^) among CD4^+^ T cells. (H) The proportion of TCMs (CD44^high^CD62L^+^) among CD4^+^ T cells. Bars indicate the mean ± SD. Based on the two-tailed Student’s *t*-test for two groups or the Tukey–Kramer test for four groups, the P-value was found to be relative to the control.

In the F2–F4 generations, we examined the proportion of germinal center (GC) B cells in the spleen. The proportion of GC B cells was found to increase as the generations progressed (Fig 2A). B cells differentiate directly into plasma cells or through GC B cells after activation. B cells with activation markers and GC B cells tended to increase, and these results suggested that long-term administration of *T*. *halophilus* No. 1 over generations enhanced B cell activation.

We also analyzed subsets of CD4^+^ T cells. First, follicular helper T (Tfh) cells involved in B cell activation were analyzed. We found that the proportion of Tfh cells did not differ in the F2 and F3 generations; however, their proportion increased in the F4 generation (Fig 2B and 2C). In addition, regulatory T (Treg) cells increased in the F3 generation (Fig 2D).

Upon activation, naive T cells differentiate into effector T cells and then give rise to two memory T cells: effector memory T cells (TEMs) and central memory T cells (TCMs). We examined the proportion of naive T cells, effector T cells, TEMs, and TCMs. In the F4 generation, wherein T cells are considered sufficiently activated by *T*. *halophilus* No. 1, the proportion of TEMs and effector T cells increased. In contrast, the proportion of TCMs and naive T cells decreased (Fig 2E–2H).

These results suggested that long-term administration of *T*. *halophilus* No. 1 in mice over generations promotes immune activation and immune tolerance.

### Long-term administration of *T*. *halophilus* No. 1 over generations increases anti-DNA IgM, suppresses autoimmunity, and decreases anti-DNA IgG, which results in autoimmunity

**Fig 3:**
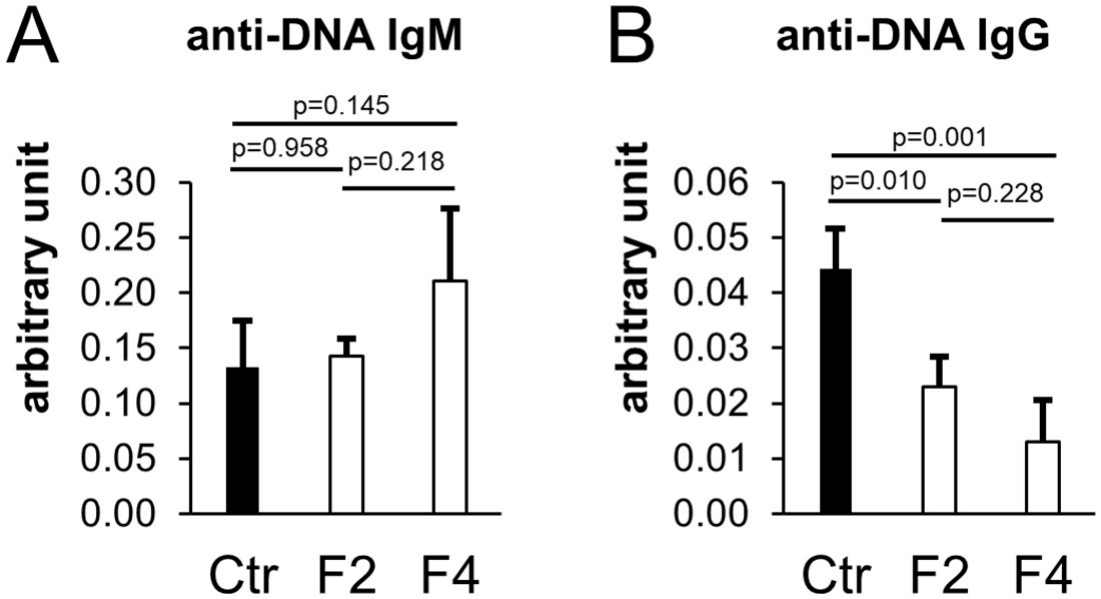
Intergenerational effects of *T*. *halophilus* No. 1 on the serum levels of anti-DNA Ig. Anti-DNA IgM and IgG serum levels were assessed. Mice (n = 4) were fed diets with or without 1% *T*. *halophilus* No. 1 until they were 16 weeks of age. Then, serum samples were obtained and analyzed. The F2 generation data were used for the control. (A) Anti-DNA IgM levels in the serum. (B) Anti-DNA IgG levels in the serum. Bars indicate mean ± SD. Based on the Tukey–Kramer test, P-value was found to be relative to the control.

Treg cells play a crucial role in suppressing autoimmunity. The serum autoantibody titer was assessed to examine whether it affected autoimmunity because Treg cells in the spleen were found to increase over generations by a continuous intake of *T*. *halophilus* No. 1. Anti-DNA IgM levels in the F2 generation showed no difference, but anti-DNA IgM levels in the *T*. *halophilus* No. 1 administration group tended to increase in the F4 generation (Fig 3A). Additionally, in the F2 generation, of the intake of *T*. *halophilus* No. 1 resulted in a decline in the anti-DNA IgG levels and decreased further in the F4 generation (Fig 3B). Serum IgM is vital to forestall autoimmunity. For instance, because serum IgM-deficient mice reportedly tended to promote the production of IgG autoantibodies [14], further immune reactions to the production of IgG autoantibodies are forestalled by IgM. DNA-specific autoantibodies of the IgG class are pathogenic and induce autoimmune symptoms such as lupus nephritis [15]. Because anti-DNA IgM levels increased and anti-DNA IgG levels decreased, long-term administration of *T*. *halophilus* No. 1 over generations was caused the suppression of autoimmunity.

## Discussion

B cells and T cells in the spleen were not found to be activated during the 2 weeks of feeding. With progress in the generation, the activation of B cells and CD4^+^ T cells was promoted by the long-term administration of *T*. *halophilus* No. 1 over generations. After the F3 generation, the Tfh cells and Treg cells increased. Furthermore, after the F2 generation, the pathogenic autoantibodies of the IgG class decreased. Therefore, immune activation and immune tolerance augmenting immunological robustness are a result of the long-term administration of *T*. *halophilus* No. 1 over generations.

The administration of miso in mice for 4 weeks increased the proportion of GC B cells and Treg cells [10]. In this study, feeding mice with diets containing *T*. *halophilus* No. 1 showed similar effects over generations. Therefore, *T*. *halophilus* No. 1 may contribute to these immunomodulatory effects of miso together with the other ingredients of miso. In contrast, although the administration of miso for 12 weeks did not increase the proportion of activated T cells or Tfh cells [10], administration of *T*. *halophilus* No. 1 over generations increased the proportion of them. Thus, consumption of miso for an extended period over generations may affect these cell populations, as is the case for *T*. *halophilus* No. 1. The administration of *T*. *halophilus* No. 1 in the F2 generation of the mice decreased the proportion of anti-DNA antibodies of IgG class, which are pathogenic. Although it is mild, miso possesses a protective effect against autoimmunity [10]. Therefore, *T*. *halophilus* No. 1 appears to account for the suppressive effect of miso on autoimmunity. These findings strongly suggested that *T*. *halophilus* No. 1 in miso adequately contributes to immunological robustness [10], possibly improving the health and longevity of Japanese people.

The effects of *T*. *halophilus* No. 1 on the immune system were shown over generations. As a transgenerational effect of miso, its consumption is inversely associated with the risk of early preterm birth [9], which is associated with autism and lifestyle-related diseases, such as metabolic syndrome, in humans [16, 17]. Furthermore, early preterm birth is associated with inflammation in animal models and humans [18, 19]. These are the indications to explain the inverse relationship between miso consumption and the risk of preterm birth. These findings suggest that daily consumption of miso and *T*. *halophilus* No. 1 may affect the health of the next generation.

Concerning the transgenerational changes caused by food intake, docosahexaenoic acid administration over three generations improves spatial learning performance and reduces anxiety-like behavior [20]. Also, prenatal exposure to probiotic *Lactobacillus lactis* affects the development of the cerebral cortex and anxiety-like behavior in the offspring [21]. Although such foods and lactic acid bacteria are found to affect behavior over generations, their mechanisms of action remain poorly understood. An epigenetic change is one of the possible mechanisms of action. Epigenetic changes have been shown to be caused by probiotics and foods [22–25]. These results suggest that a long-term administration of miso or *T*. *halophilus* No. 1 induces such changes.

Collectively, some effects of *T*. *halophilus* No. 1 on the immune system appeared only over generations. Studies on long-term administration over generations in mice may aid in determining the precise impacts of daily food on human health.

## Supporting information

S1 Fig The short-term effects of *T*. *halophilus* No. 1 on B cells and T cells. The proportion of spleen B cells and T cells in mice was examined. Mice (n = 12) were fed a diet with or without 1% *T*. *halophilus* No. 1 for 2 weeks. Then, the spleen samples were obtained and analyzed by flow cytometry. (A) The proportion of B cells among the total lymphocytes. (B) The proportion of CD4^+^ T cells among the total lymphocytes. (C) The proportion of CD86^+^ among B cells. (D) The proportion of CD69^+^ among B cells. (E) The proportion of CD69^+^ among CD4^+^ T cells. Bars indicate the mean ± SD. P-value is relative to the control based on the two-tailed Student’s *t*-test.
(TIF)

S2 Fig The profiles of total and activated B cells and T cells. Examples of the measurement of flow cytometry in Fig 1 are indicated. (A) B220 and CD4. (B) CD4 and CD8a. (C) B220 and CD86. (D) B220 and CD69. (E) CD4 and CD69.
(TIF)

S3 Fig The profiles of subsets of CD4^+^ T cells. Examples of the measurement of flow cytometry in Fig 2 are indicated. (A) PD-1 and CXCR5 in CD4^+^. (B) PD-1 and ICOS in CD4^+^. (C) CD127 and CD25 in CD4^+^. (D) CD127 and CTLA4 in CD4^+^CD127^−^CD25^+^. (E) CD44 and CD62L in CD4^+^.
(TIF)
