# Supplementary figures and images for: Long-term administration of Tetragenococcus halophilus No. 1 over generations affects the immune system of mice
Source: PLoS One. 2022 Apr 26;17(4):e0267473. doi: 10.1371/journal.pone.0267473 (PMC9041805; doi:10.1371/journal.pone.0267473)

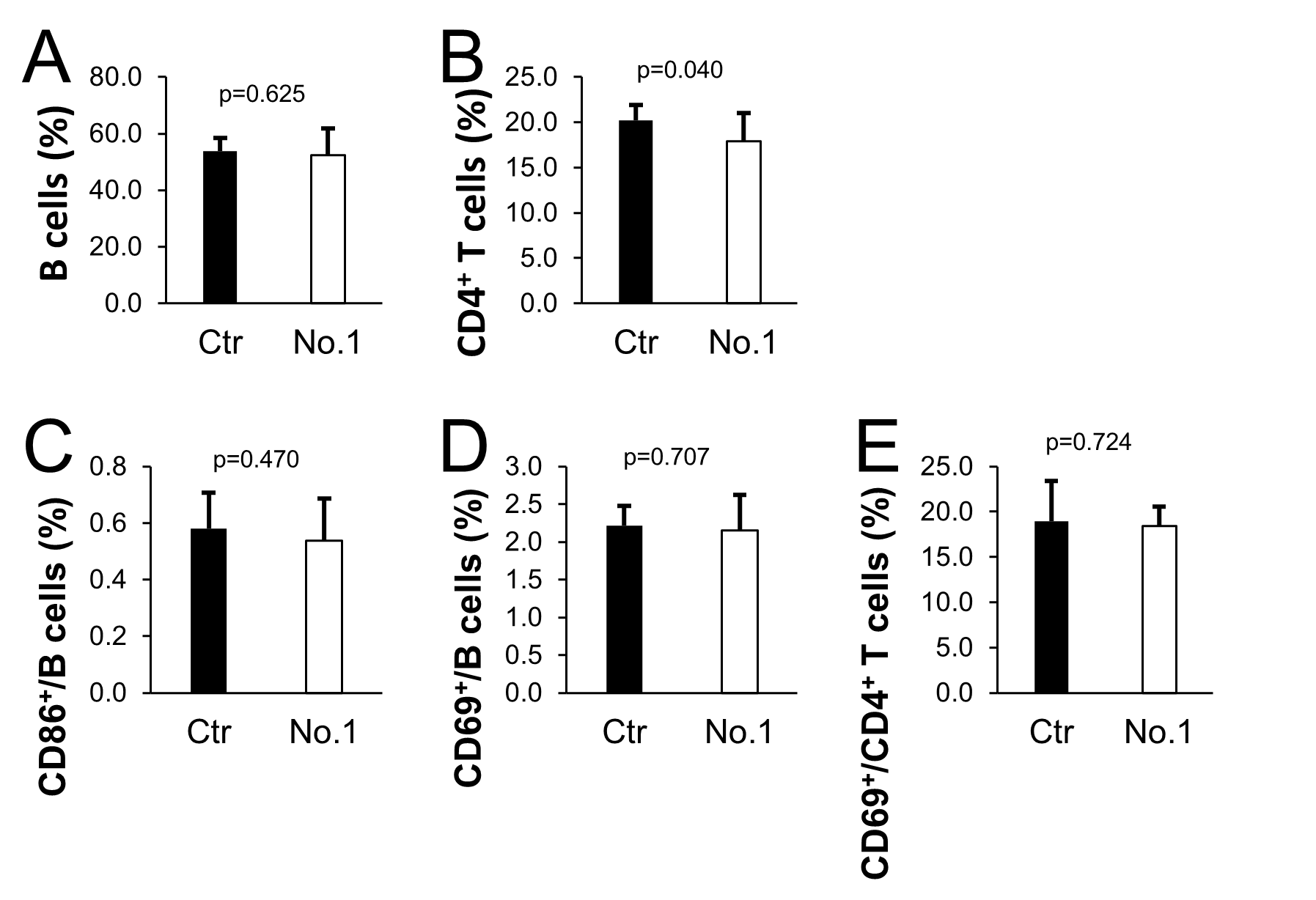

Supplement: S1 Fig — The proportion of spleen B cells and T cells in mice was examined. Mice (n = 12) were fed a diet with or without 1% T. halophilus No. 1 for 2 weeks. Then, the spleen samples were obtained and analyzed by flow cytometry. (A) The proportion of B cells among the total lymphocytes. (B) The proportion of CD4+ T cells among the total lymphocytes. (C) The proportion of CD86+ among B cells. (D) The proportion of CD69+ among B cells. (E) The proportion of CD69+ among CD4+ T cells. Bars indicate the mean ± SD. P-value is relative to the control based on the two-tailed Student’s t-test. (TIF) [file pone.0267473.s001.tif]

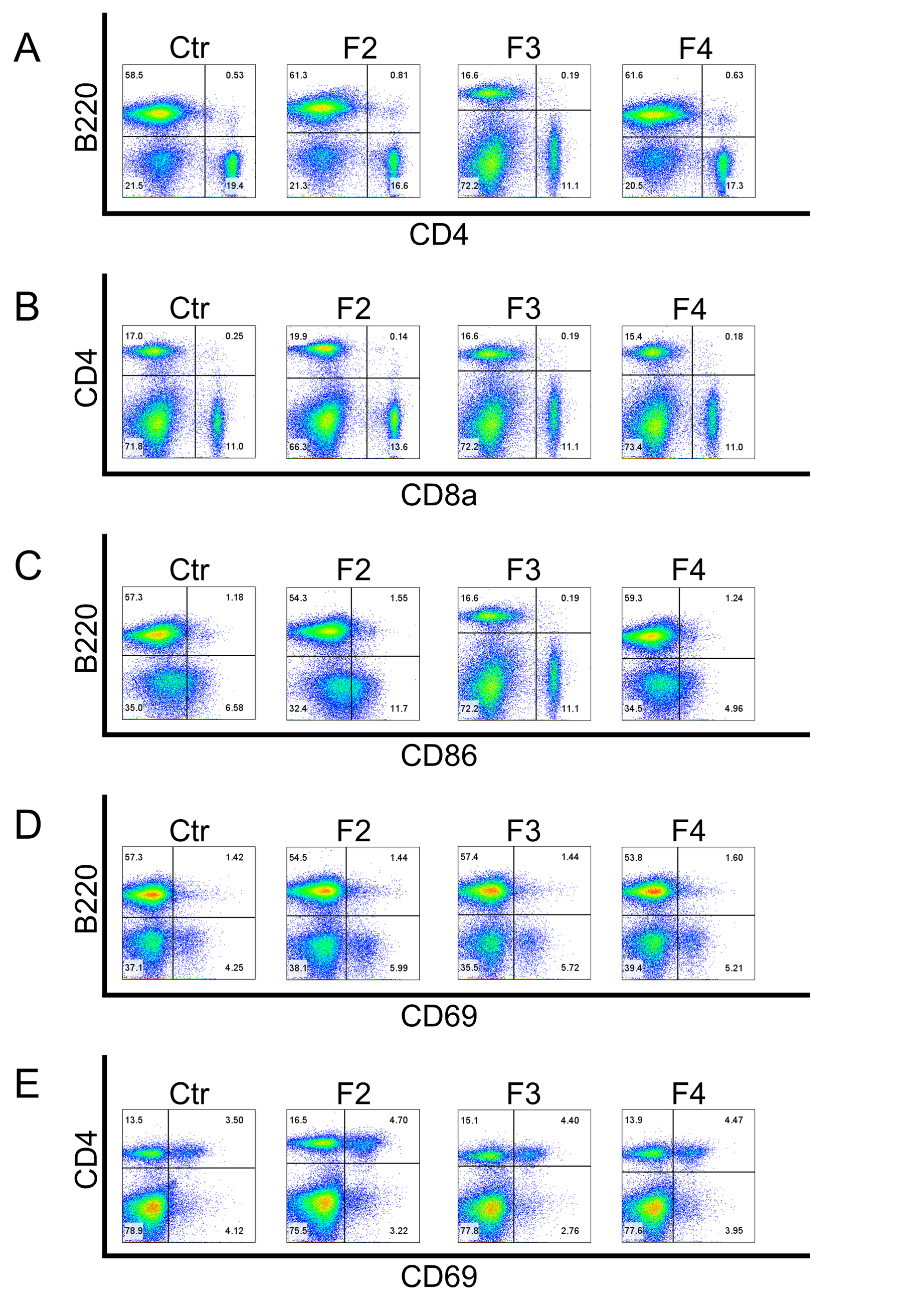

Supplement: S2 Fig — Examples of the measurement of flow cytometry in Fig 1 are indicated. (A) B220 and CD4. (B) CD4 and CD8a. (C) B220 and CD86. (D) B220 and CD69. (E) CD4 and CD69. (TIF) [file pone.0267473.s002.tif]

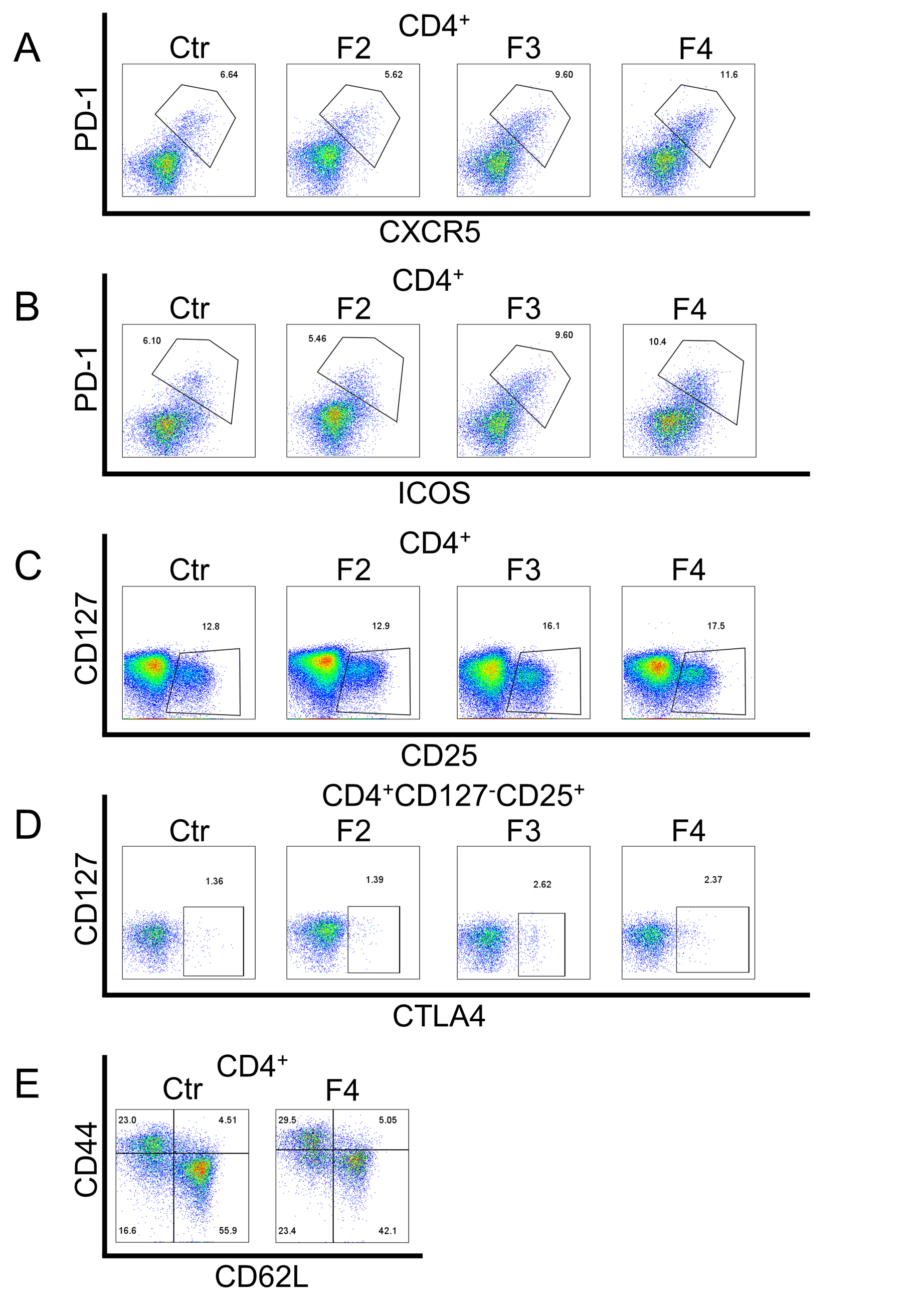

Supplement: S3 Fig — Examples of the measurement of flow cytometry in Fig 2 are indicated. (A) PD-1 and CXCR5 in CD4+. (B) PD-1 and ICOS in CD4+. (C) CD127 and CD25 in CD4+. (D) CD127 and CTLA4 in CD4+CD127−CD25+. (E) CD44 and CD62L in CD4+. (TIF) [file pone.0267473.s003.tif]
